# Amorphous boron nitride as a low-*k* dielectric for next-generation BEOL interconnects

**DOI:** 10.1186/s40580-026-00579-5

**Published:** 2026-10-01

**Authors:** Jong Won Baek, Ju-Hyun Jung, Hyeong-Seok Kim, Junho Kim, Dong-Hyun Kim, Jangyup Son, Sangyul Ha

**Affiliations:** 1 Department of Mechanical Engineering, POSTECH, Pohang, 37673 Republic of Korea; 2 Institute of Advanced Composite Materials, Korea Institute of Science and Technology (KIST), Wanju-gun, Jeonbuk 55324 Republic of Korea; 3 Department of Materials Science and Engineering, Korea University, Seoul, 02841 Republic of Korea; 4 Department of Electrical and Electronic Engineering, KAIST, Daejeon, 34141 Republic of Korea; 5 SKKU Advanced Institute of Nanotechnology (SAINT), Sungkyunkwan University, Suwon, 16419 Republic of Korea; 6 Nanoscience and Technology, KIST School, University of Science and Technology, Seoul, 02792 Republic of Korea; 7 Division of Semiconductor Engineering, Myongji University, Yongin, Gyonggi 17058 Republic of Korea

**Keywords:** Amorphous boron nitride, Low-*k* dielectric, BEOL interconnects, Hybrid bonding

## Abstract

As back-end-of-line (BEOL) scaling pushes porous low-*k* dielectrics toward their reliability limits, nonporous dielectrics that combine a low dielectric constant (*k*) with mechanical, thermal, and interfacial robustness are increasingly needed. Among nonporous low-*k* candidates, amorphous boron nitride (aBN) has attracted attention for advanced BEOL interconnects owing to its low-*k* value (*k* ≈ 2) and high mechanical robustness, as reflected by a Young’s modulus of ~50 GPa. In this review, we discuss aBN from a BEOL process-integration perspective, focusing on the origin of its low-*k* behavior, process-dependent structure–property relationships, and its potential roles in BEOL interconnects and hybrid-bonding interfaces. Finally, we discuss remaining issues and future directions for validating aBN as a nonporous low-*k* dielectric/interface material under BEOL and hybrid-bonding integration.

**Graphical abstract:**

**Figure:**
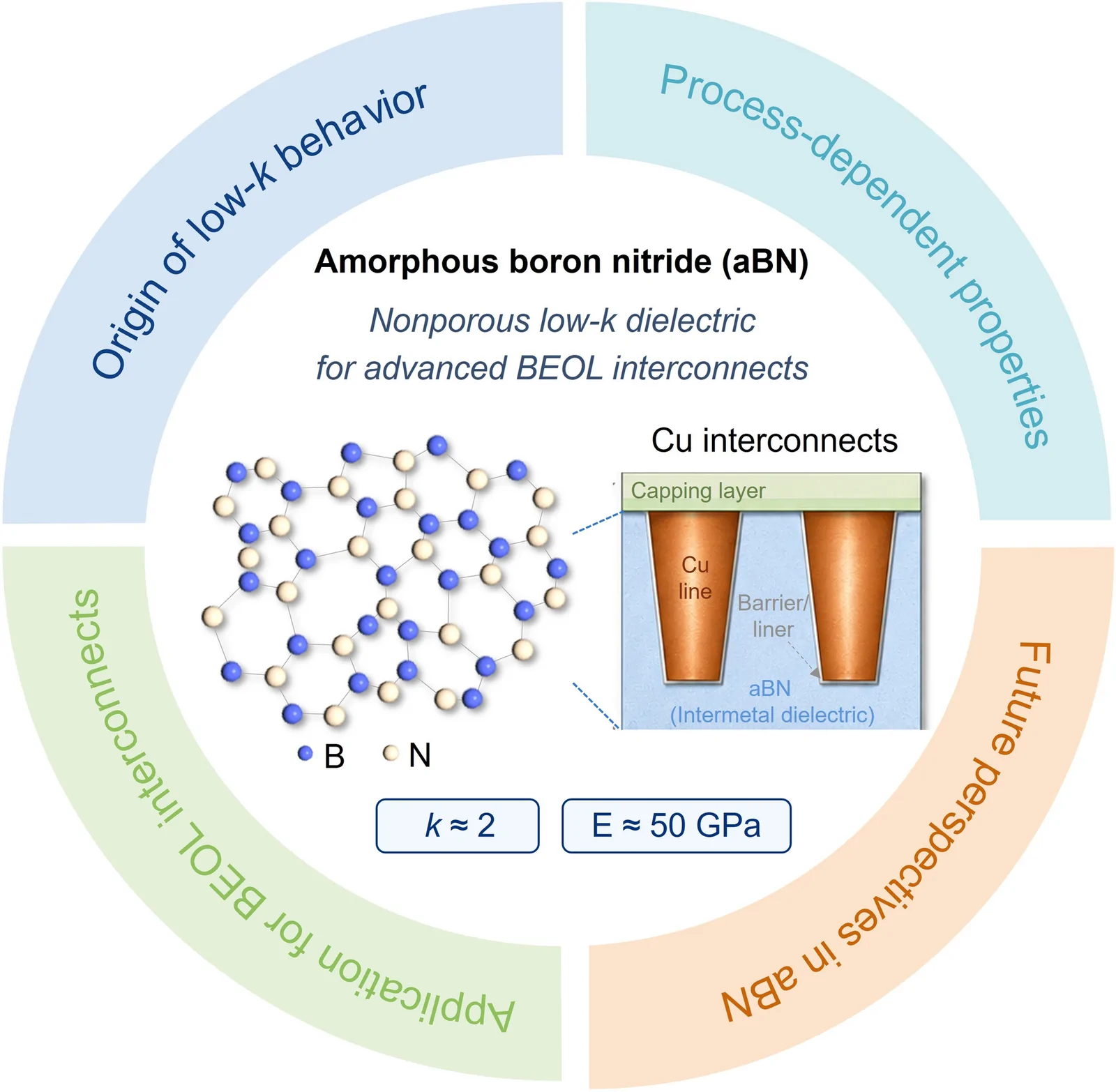

## Introduction

The development of semiconductor technology has been driven by the demand for higher integration density, smaller device dimensions, and faster data processing [1, 2]. This demand has become critical with the rapid expansion of artificial intelligence and high-performance computing. Traditionally, device performance has been improved mainly through front-end-of-line (FEOL) scaling, including linewidth reduction and layout optimization. However, as FEOL scaling faces physical and lithographic limitations, further performance improvement increasingly depends on innovations in the back-end-of-line (BEOL) region [3–5].

**Fig. 1:**
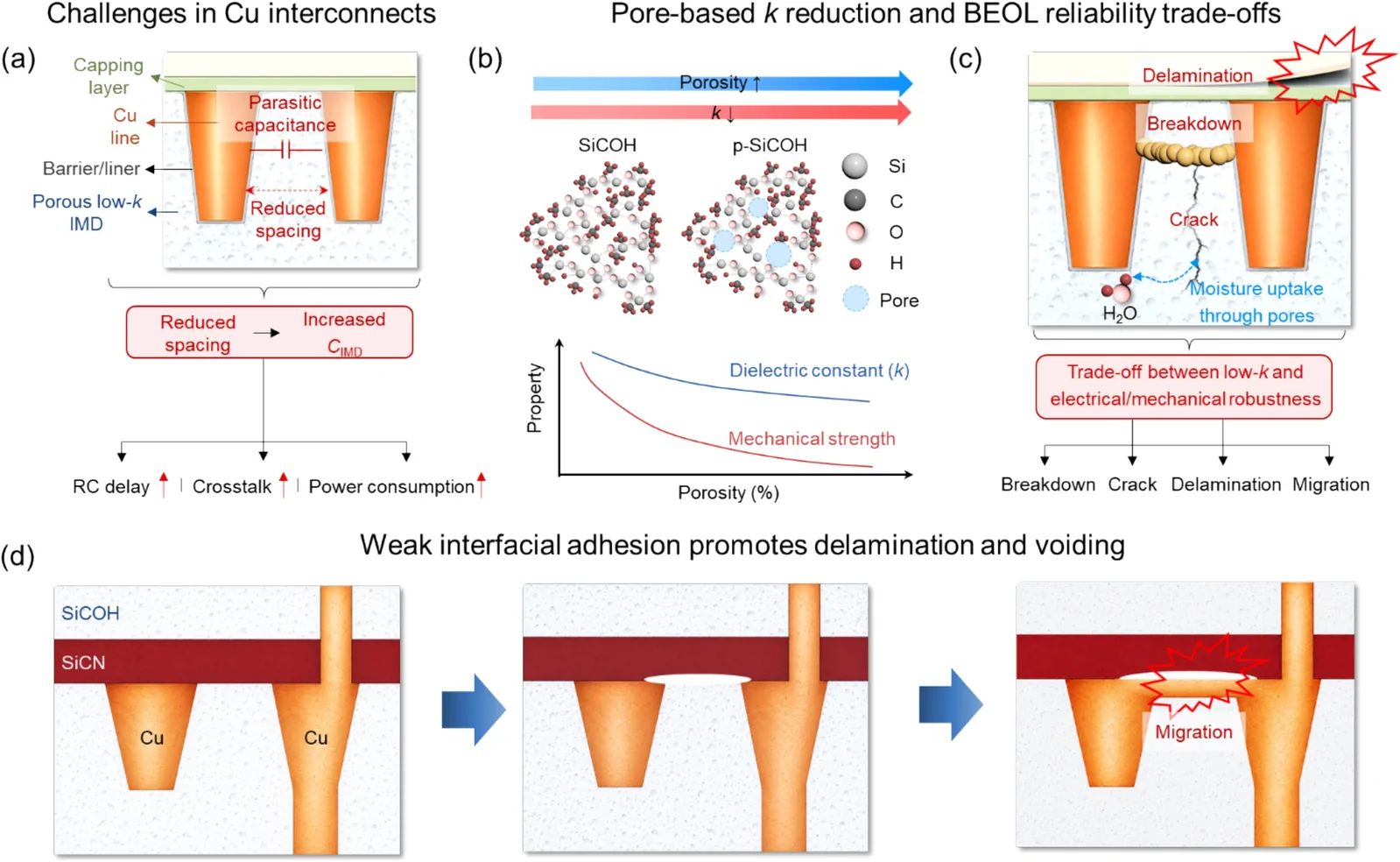
Materials and reliability challenges in advanced BEOL interconnects. **a** Schematic illustration of BEOL metal interconnects separated by intermetal dielectrics. **b** Porosity-based *k* reduction strategy represented by porous SiCOH. **c** Schematic of reliability issues in porous low-*k* dielectrics. **d** Thermo-mechanical stress and stress migration in BEOL interconnects

In BEOL interconnects, densely packed metal lines are separated by intermetal dielectrics (IMDs), and the capacitance between adjacent interconnects becomes a major contributor to RC delay and capacitive crosstalk (Fig. 1a) [6–8]. At advanced technology nodes, narrower interconnect widths increase line resistance, while reduced line-to-line spacing enhances capacitive coupling, further increasing RC delay and limiting signal propagation speed. Therefore, lowering the dielectric constant (*k*) of IMDs has become critical for improving the electrical performance of interconnect structures [9]. To meet this requirement, industrially adopted IMDs have evolved from fluorinated SiO_2_ to porous SiCOH, with the aim of continuously reducing interconnect capacitance as technology nodes scale [10]. Among these, porous low-*k* dielectrics, represented by porous SiCOH, have been developed, in which *k* is reduced mainly by incorporating nanoscale pores filled with air or vacuum (*k* ≈ 1) into the dielectric matrix (Fig. 1b) [4, 11]. Previous studies have attempted to reduce *k* by increasing the pore volume fraction to about 60% [12]. However, this porosity-driven reduction in *k* is accompanied by an intrinsic trade-off between *k* and electrical/mechanical robustness [13, 14]. The introduction of pores increases moisture uptake and can provide leakage or Cu-migration pathways, resulting in reliability issues such as dielectric breakdown, crack formation, and interfacial delamination (Fig. 1c) [15–20]. Moreover, continued scaling of IMD thickness and interconnect spacing imposes increasingly stringent requirements on dielectric and mechanical stability, making dielectric integration has become increasingly challenging because low-k properties and mechanical reliability must be maintained throughout BEOL processes, including plasma treatment, etching, thermal processing, and chemical mechanical polishing (CMP).

Therefore, next-generation BEOL interconnects require dielectric materials that can simultaneously provide low-*k*, sufficient mechanical robustness, strong interfacial adhesion, and metal diffusion-barrier functionality. This has motivated increasing interest in nonporous low-*k* dielectrics that can avoid the pore-induced degradation mechanisms of porous low-*k* materials [21–23]. Among the emerging candidates, amorphous boron nitride (aBN) has recently attracted attention [24] because it offers a fundamentally different low-*k* design strategy based on an amorphous B–N network rather than pore formation. In addition, ultrathin aBN films have been reported to exhibit low-*k*, high breakdown strength, and metal diffusion-barrier characteristics, suggesting their possible use as an IMD or bonding-interface dielectric [24, 25].

While the comprehensive synthesis methodologies, foundational physical properties, and broad device applications of aBN have been excellently reviewed in recent literature [26], this review specifically shifts the focus away from intrinsic characteristics to establish a highly specialized, application-oriented perspective dedicated exclusively to advanced semiconductor manufacturing. To minimize overlap with previous publications, we have excluded general explanations regarding synthesis and basic physics. Instead, this review discusses aBN as a low-*k* dielectric for next-generation BEOL interconnects. First, the limitations of conventional low-*k* dielectrics and bonding layers are summarized. The low-*k* origin of aBN is then discussed in terms of its amorphous network and bonding characteristics. Subsequently, process-dependent structure–property relationships are reviewed, with emphasis on precursor chemistry, hydrogen incorporation, growth rate, and gas species. Finally, BEOL and hybrid bonding applications are examined, together with remaining future work related to dielectric reliability, interfacial adhesion, moisture stability, wafer warpage, process integration, and the extension of aBN toward hybrid-bonding interfaces.

## From porous to nonporous low-*k* dielectrics for BEOL interconnects

### Limitations of pore-based low-*k* dielectrics

BEOL integration requires strong interfacial adhesion between metal lines and low-*k* dielectrics, [15] because these interfaces are repeatedly exposed to thermo-mechanical stress during fabrication and reliability testing. In BEOL stacks, which consist of metals and dielectric layers with different coefficients of thermal expansion, this mismatch can increase the risk of interfacial delamination and crack formation. Such stress can be further amplified at structural and material discontinuities, including T-shaped junctions, line bends, via/contact regions, and Cu/W interfaces. These local stress gradients provide a driving force for stress migration (SM) [27, 28]. During SM, vacancies or voids tend to accumulate in tensile-stress regions, whereas metal atoms migrate toward compressive-stress regions. As a result, SM can lead to void formation, metal depletion, and ultimately reliability degradation in BEOL interconnects (Fig. 1d) [29]. If porous low-*k* dielectrics have weak adhesion to adjacent metal lines, such stress induces interfacial delamination and promotes SM and stress-induced voiding, in which stress-driven Cu transport and vacancy accumulation lead to void formation, increased line resistance, and open-circuit failure [30, 31]. Therefore, new low-*k* dielectric materials must simultaneously satisfy the requirements of mechanical robustness, strong interfacial adhesion, and diffusion-barrier functionality for next-generation BEOL interconnect technologies.

### Nonporous low-*k* design principle of aBN

**Fig. 2:**
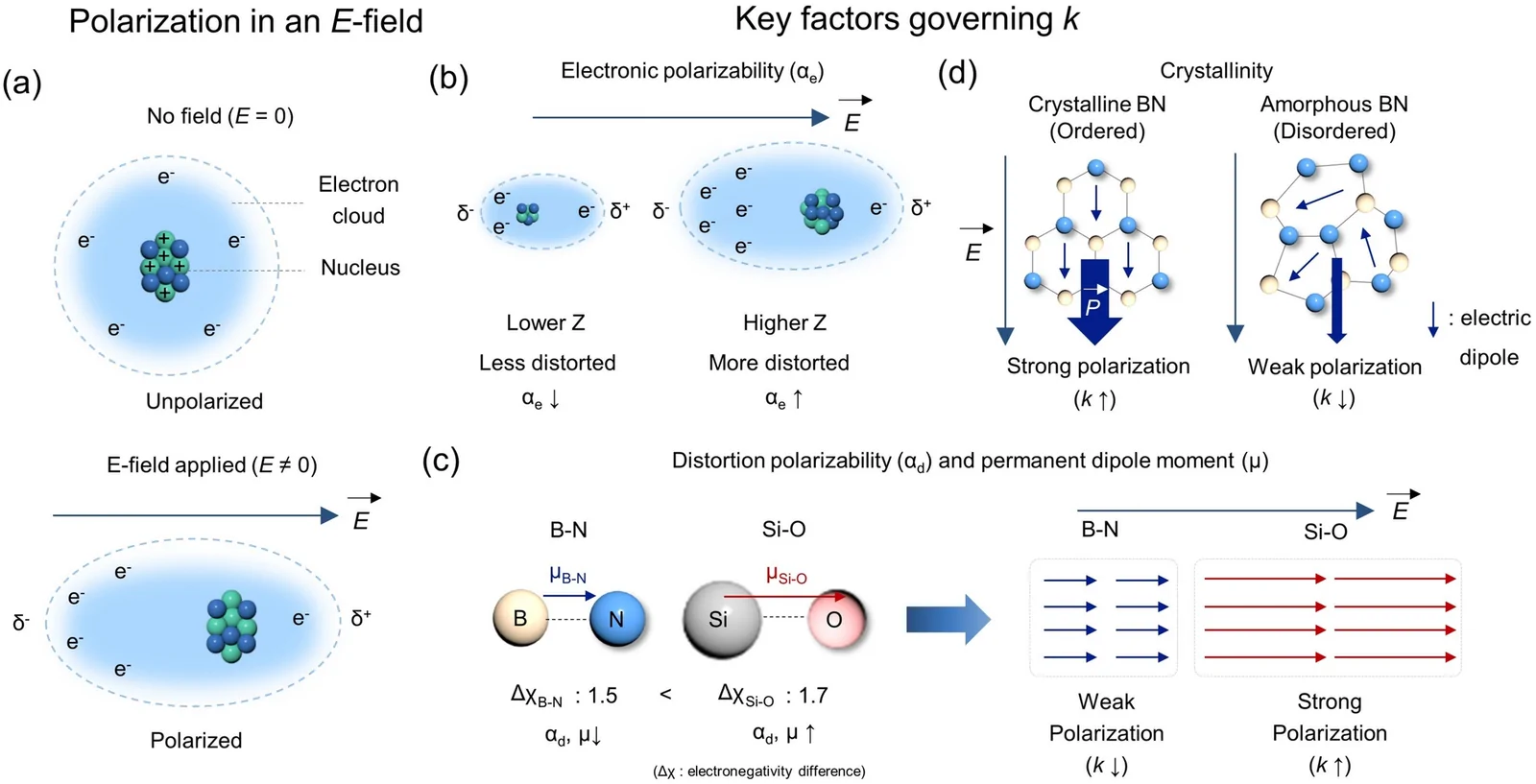
Fundamental origin of low-*k* behavior in nonporous dielectrics. a Schematic of dielectric polarization under an external electric field, showing the nucleus surrounded by the electron cloud. **b** Atomic-number-dependent electronic polarizability. **c** The effect of bond electronegativity difference on distortion polarizability and permanent dipole moment. **d** Schematic illustration of the effect of crystallinity on dielectric polarization, comparing the field-induced polarization responses of crystalline BN and aBN

To design nonporous low-*k* materials, it is necessary to identify the polarization mechanisms that determine *k*. When an external electric field is applied, *k* reflects how strongly the material becomes polarized in response to the field (Fig. 2a). In the Debye equation, this polarization can be expressed as the sum of electronic polarizability, distortion polarizability, and orientational polarization: [32]

$$\frac{k-1}{k+2}= \frac{4\pi }{3}N\left({\alpha}_{e}+{\alpha}_{d}+\frac{{\mu }^{2}}{3{k}_{B}T}\right)$$

where *k* is the dielectric constant, *N* is the density of dipoles, *α*_e_ is the electronic polarizability, *α*_d_ is the distortion polarizability, *μ* is the permanent dipole moment, *k*_B_ is the Boltzmann constant, and *T* is the absolute temperature. This relationship indicates that *k* can be reduced by suppressing each polarization contribution. In general, atoms with lower atomic numbers have smaller and less deformable electron clouds, resulting in lower electronic polarizability (Fig. 2b). In addition, bonds with smaller electronegativity differences exhibit weaker bond polarity, which reduces distortion polarizability and permanent dipole moments (Fig. 2c). For example, the electronegativity difference of the B–N bond (Δχ = 1.5) is smaller than that of the Si–O bond (Δχ = 1.7), indicating weaker bond polarity. Under an external electric field, weaker B–N bond polarity leads to less field-induced bond distortion and a smaller permanent dipole contribution than that of the highly polar Si–O bond. As a result, B–N-based networks can exhibit weaker polarization and a lower dielectric constant than Si–O-based networks. Therefore, materials composed of low-polarizability atoms and weakly polar bonds can suppress electric-field-induced polarization, leading to a lower *k* [24, 33].

Based on these design principles, the low-*k* behavior of aBN can be understood as arising from its intrinsic bonding chemistry. This behavior can be attributed to two key factors. First, B and N have lower atomic numbers than Si, resulting in reduced *α*_e_. Second, the smaller electronegativity difference of the B–N bond compared with the Si–O bond reduces bond polarity, thereby suppressing ${\alpha}_{d}$ and *μ*. As a result, aBN exhibits weak field-induced polarization.

In addition, crystallinity can further influence the dielectric response (Fig. 2d). In crystalline materials, long-range structural order can facilitate dipole alignment under an external electric field, resulting in a stronger field-induced polarization and thereby increasing *k*. In contrast, amorphous structures lack long-range structural order and exhibit randomly distributed local bonding orientations. Consequently, dipole alignment under an applied electric field is limited, resulting in a weaker field-induced polarization and thereby lowering *k*. Thus, although both crystalline BN and aBN are predominantly composed of B–N bonds, their different degrees of structural order can lead to substantially different dielectric responses. Therefore, among BN structures, an sp^2^-rich bonding configuration combined with an amorphous structure is favorable for achieving a low-*k*. Because this low-*k* behavior is achieved without pore formation, aBN can maintain a dense dielectric network and high mechanical robustness, as reflected by its reported Young’s modulus of ~50 GPa, which is much higher than that of porous SiCOH (~ 4.3 GPa) [26, 34, 35].

## Process-condition-dependent mechanical/dielectric properties

For BEOL integration, understanding how processing conditions affect the dielectric and mechanical properties of dielectric materials is essential. In this section, SiCOH and SiCN are considered as two distinct reference systems for different BEOL applications. Porous SiCOH represents a conventional low-*k* IMD, whereas SiCN is commonly employed as a capping, diffusion-barrier, and bonding dielectric. Because aBN has been proposed not only as a low-*k* IMD but also as an ultrathin barrier or bonding/interface dielectric, both SiCOH and SiCN provide relevant benchmarks for evaluating its process-dependent properties and potential applications. However, comparable process–property relationships remain less systematically established for aBN. Therefore, this section briefly summarizes the process-dependent characteristics of these conventional BEOL dielectrics before discussing the current understanding of aBN and the key process variables that require further investigation.

### Process-dependent properties of conventional low-*k* dielectrics

In SiCOH, *k* and mechanical robustness are mainly governed by two factors: methyl (CH_3_) termination and porosity. Terminal Si–CH_3_ groups reduce bond polarizability and decrease the density of polarizable Si–O bonds, leading to lower *k* [5]. However, excessive Si–CH_3_ incorporation breaks Si–O bonding and introduces terminal organic groups, increasing free volume and reducing the elastic modulus [5]. Therefore, to further reduce *k*, sacrificial porogens are incorporated during deposition and subsequently removed by UV curing to generate nanopores [6, 36]. As a result, porous SiCOH becomes more vulnerable to plasma-induced damage and moisture uptake [37–40]. During etch and ash processes, reactive plasma species can penetrate the porous film, break Si–CH_3_/Si–C bonds, and form hydrophilic Si–OH bonds [41, 42]. These Si–OH groups absorb moisture, increasing film polarizability and raising *k* [5, 6].

These two factors are strongly influenced by deposition and post-treatment conditions, which ultimately determine the final dielectric and mechanical properties of SiCOH. A lower deposition temperature can increase –CH and –CH₃ incorporation [43], thereby reducing *k* but also lowering elastic modulus [44]. Plasma power changes the degree of precursor decomposition and film densification; higher power generally increases density and modulus but can also remove methyl groups and increase *k* [45]. The flow rate ratio of matrix precursor to porogen determines the pore volume fraction and therefore directly controls the *k*–modulus trade-off [46]. UV curing removes porogen to form pores while simultaneously reconstructing the Si–O–Si network and partially recovering mechanical strength. However, excessive UV curing can re-densify or oxidize the film, leading to an increase in *k* [5, 35].

Unlike porous SiCOH, SiCN is generally used as a relatively dense capping, diffusion-barrier, or bonding dielectric in BEOL integration. Its dielectric and mechanical properties are primarily governed by the Si–C/Si–N bond ratio, hydrogen incorporation, and film density. Increasing carbon-rich Si–C bonding can reduce *k* because Si–C bonds are less polar than Si–N bonds, whereas hydrogen incorporation can modify film density and network connectivity. These bonding characteristics are strongly affected by deposition conditions, including precursor composition, plasma power, and deposition temperature. Thus, SiCN provides a reference for evaluating the process-dependent properties of aBN, particularly for its potential use as a dense capping, barrier, or bonding/interface dielectric.

### Process-dependent properties of aBN

**Fig. 3:**
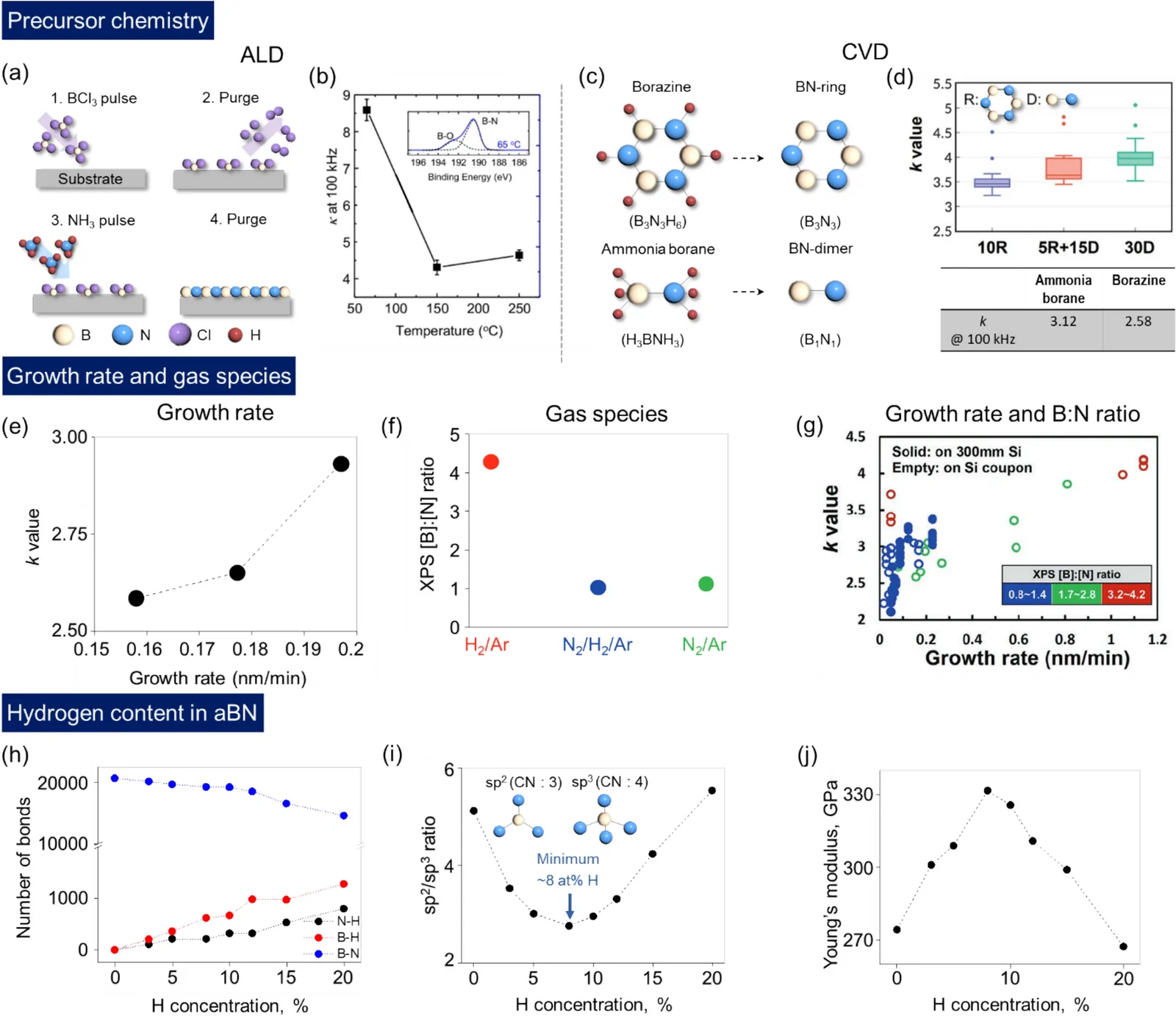
Process-dependent dielectric and mechanical properties of aBN. **a** Schematic of ALD aBN deposition using BCl_3_ and NH_3_ precursors. **b** Effect of oxygen incorporation on the *k* value of ALD-grown aBN [47]. **c** Representative CVD single-source BN precursors: borazine for BN-ring network formation and ammonia borane for dimer-rich BN network formation [34]. **d** Precursor-dependent *k* values of CVD-grown aBN [34]. **e** Growth-rate-dependent *k* of aBN [34]. **f**, **g** Effects of **f** gas species [34] and **g** B:N stoichiometry on the *k* value of aBN [34]. **h**–**j** Hydrogen-content-dependent changes in **h** number of N–H, B–H and B–N bonds [53], **i** sp^2^/sp^3^ hybridization ratio [53] and **j** the Young’s modulus of aBN [53]. **b** Adapted from ref. [47] under the terms of the Creative Commons Attribution License (CC BY 4.0). Copyright 2024, Springer Nature. **c**, **g** Adapted with permission from ref. [34]. Copyright 2022 Wiley–VCH GmbH

Precursor chemistry is a key parameter that influences the dielectric properties of aBN. Representative precursors include BCl_3_ as a B precursor combined with NH₃ as an N precursor for ALD processes, as well as CVD single-source BN precursors such as borazine and ammonia borane [34, 47–50]. In ALD, BCl_3_ and NH_3_ are alternately supplied, terminated through self-limiting surface reactions, and purged during each ALD cycle (Fig. 3a) [47]. Although this process enables conformal aBN deposition, incomplete ligand elimination can leave residual B–Cl bonding in the film. These residual species can be oxidized during the ALD process to form B–O bonds, which increase film polarizability and thereby increase *k* (Fig. 3b). Furthermore, increasing the deposition temperature can reduce residual B–Cl bonding by promoting ligand removal, but the reduction in *k* remains limited if oxygen is incorporated into the aBN film.

For CVD-grown aBN, the molecular structure of single-source BN precursors determines the resulting B–N network (Fig. 3c) [34]. This difference affects *k* because ring-based structures have a higher coordination environment than dimer-based structures, which suppresses electron delocalization and reduces polarization. In contrast, dimer-rich structures promote electron delocalization, resulting in stronger polarization and a higher *k*. Consistent with this trend, simulation results showed that aBN structures composed of 10 rings exhibited lower *k* values than those composed of 30 dimers (Fig. 3d). Experimentally, borazine-derived aBN also showed a lower *k* value of 2.58 than ammonia-borane-derived aBN with *k* = 3.12, supporting the trend predicted by the simulation. However, this ring–dimer description does not fully represent the actual decomposition chemistry of ammonia borane, because ammonia borane can form intermediates such as borazine during decomposition. Therefore, the dimer-based model should be regarded as a simplified structural representation.

In addition, lower growth rates provide sufficient structural relaxation time for fused B–N ring units to form short-range stacked networks with lower net dipole moments, resulting in lower *k* values (Fig. 3e) [34]. Process gas species affect the B:N stoichiometry of aBN films (Fig. 3f); N_2_-containing gases help maintain a near-stoichiometric B:N ratio, whereas H_2_/Ar-based conditions tend to produce B-rich aBN films containing borides and boron clusters, which increase *k* (Fig. 3g) [34].

The bonding configuration of aBN is also an important parameter governing its dielectric and mechanical properties. aBN can exhibit diverse bonding configurations, including sp^1^, sp^2^, and sp^3^ bonding. A recent study suggests that sp^2^-dominated aBN exhibits a lower *k*, whereas sp^3^-rich structures and sp^1^-coordinated defects are associated with higher *k* values^33^. The higher *k* of sp^3^-rich structures was mainly attributed to their higher atomic density, which increases the number of polarizable atoms and bonds per unit volume. In contrast, sp^1^-coordinated sites can induce disorder-induced mid-gap states, which can further increase k by enabling low-energy electronic transitions to or from delocalized states. Furthermore, the reported *k* values of approximately 3.8 for sp^2^-bonded hBN [51] and 7.1 for sp^3^-bonded cBN [52] are consistent with the above theoretical trend, indicating that an sp^2^-rich bonding configuration is beneficial for achieving a lower *k* in BN.

Interestingly, hydrogen content can tune the mechanical properties of aBN by modifying its local bonding structure. Atomistic simulations showed that H incorporation changes the sp^2^/sp^3^ hybridization ratio and chemical bonding network of aBN (Fig. 3h–j) [53]. At moderate H concentrations, the formation of B–H and N–H bonds reduces the number of low-coordinated sites (Fig. 3h) and promotes a more compact sp^3^-rich network (Fig. 3i). An increased sp^3^ fraction, together with a reduction in sp^1^-coordinated sites and dangling bonds, leads to a denser and more highly connected aBN network, thereby improving its mechanical robustness [53]. As a result, the sp^2^/sp^3^ ratio is minimized at approximately 8 at% H, where the sp^3^ fraction is maximized, and the Young’s modulus increases from 270.11 GPa for pure aBN to 332.21 GPa (Fig. 3j). However, excessive H incorporation disrupts the continuous B–N network by terminating B/N atoms with H, which causes low-coordinated sites to re-emerge. This reduces the sp^3^ fraction and degrades the Young’s modulus. These results highlight a trade-off in the bonding structure of aBN: an sp^2^-rich network is advantageous for achieving a low-*k*, whereas increased sp^3^ coordination can improve mechanical robustness. Therefore, rather than simply maximizing either sp^2^ or sp^3^ bonding, the bonding configuration of aBN should be optimized to simultaneously maintain a low-*k* and sufficient mechanical integrity for BEOL interconnect applications. The optimal sp^2^/sp^3^ ratio that satisfies both requirements, however, has not yet been established. Thus, H should be controlled as a key process-dependent structural modifier. In addition, C incorporation has been predicted to enhance the stability and Young’s modulus of aBN by increasing sp^3^ bonding, but further studies are needed to clarify how H and C contents simultaneously affect the dielectric constant, leakage current, and breakdown reliability of aBN [33].

## Application for BEOL interconnects

**Fig. 4:**
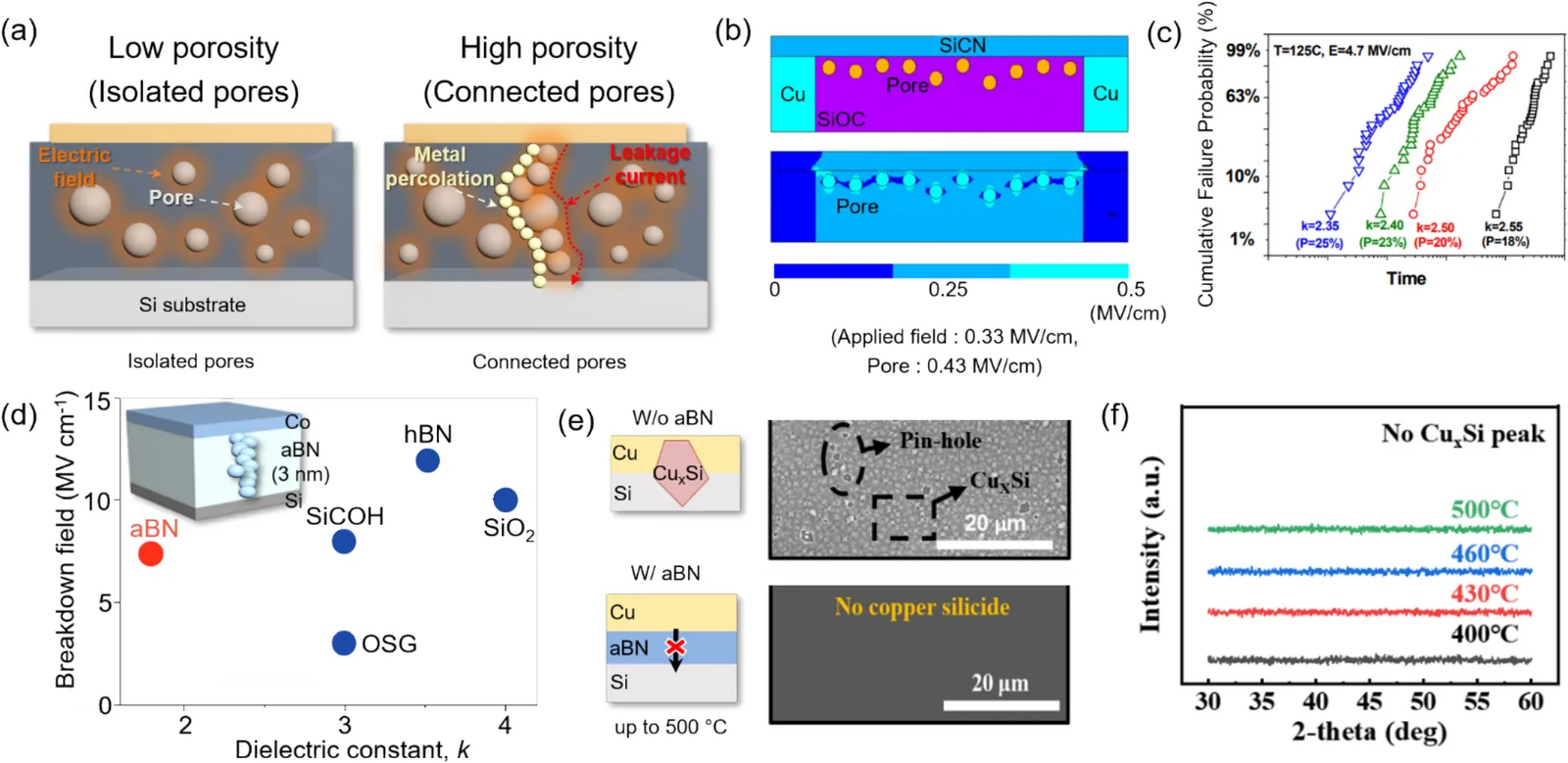
Pore-assisted electrical and Cu-diffusion failure in porous low-*k* dielectrics and the potential role of aBN. **a** Schematic illustration of pore-connectivity-dependent failure pathways in porous low-*k* dielectrics. **b** Electric-field simulation of porous SiCOH, showing local field accumulation around pores [55]. **c** TDDB failure distribution of porous low-*k* dielectrics with different pore fractions [55]. **d** Comparison of breakdown field between conventional low-*k* dielectrics and aBN [24]. **e** Cross-sectional SEM images [25] and **f** XRD patterns showing the Cu-diffusion-blocking behavior of aBN in Cu/aBN/Si structures after thermal annealing [25]. **b, c** Adapted with permission from ref. [55]. Copyright 2009 IEEE. **e**, **f** Adapted with permission from ref. [25]. Copyright 2023 IEEE

In porous low-*k* dielectrics, dielectric reliability is strongly governed by pore connectivity, which evolves as porosity increases [13]. At low porosity, pores are spatially isolated, so the electric field is localized around pores, and leakage pathways remain discontinuous (Fig. 4a) [54]. However, as porosity increases, the internal pore surfaces can provide pathways where the electric field is locally concentrated. These connected pore surfaces also facilitate moisture penetration and Cu-ion migration, thereby promoting the formation of leakage and metal-percolation pathways and accelerating dielectric breakdown [55].

Electric field simulation supports the reliability limitation of porous low-*k* dielectrics. In porous SiCOH, the electric field is more strongly concentrated around the pores than in the surrounding matrix (Fig. 4b) [55]. This local field concentration becomes more severe as the pore fraction increases, providing electrically weak regions for leakage-path formation. Consistently, Time Dependent Dielectric Breakdown (TDDB) measurements show that the time-to-breakdown decreases with increasing pore ratio (Fig. 4c) [55]. In contrast, aBN can maintain a comparable breakdown field with a lower *k* value than SiCOH (Fig. 4d) [24]. This suggests that aBN can overcome the intrinsic trade-off between pore ratio and electrical reliability in porous low-*k* dielectrics [24, 25].

When aBN was inserted between Cu and Si and annealed up to 500 °C, neither Cu–Si alloy formation in SEM images nor Cu silicide-related peaks in XRD patterns were observed (Fig. 4e, f) [25], indicating that aBN can suppress Cu diffusion under high-temperature conditions. However, whether aBN is intrinsically superior to conventional SiCOH as a Cu barrier remains unresolved, and the atomistic origin of its diffusion-blocking behavior is still unclear [56]. At present, this behavior may be attributed to the relatively high density and compact atomic network of aBN, which can hinder the formation of continuous metal- or ion-migration pathways. Further studies combining time-to-failure measurements, activation-energy extraction, and atomistic simulations are required to compare aBN with SiCOH and clarify the Cu-diffusion mechanism.

**Fig. 5:**
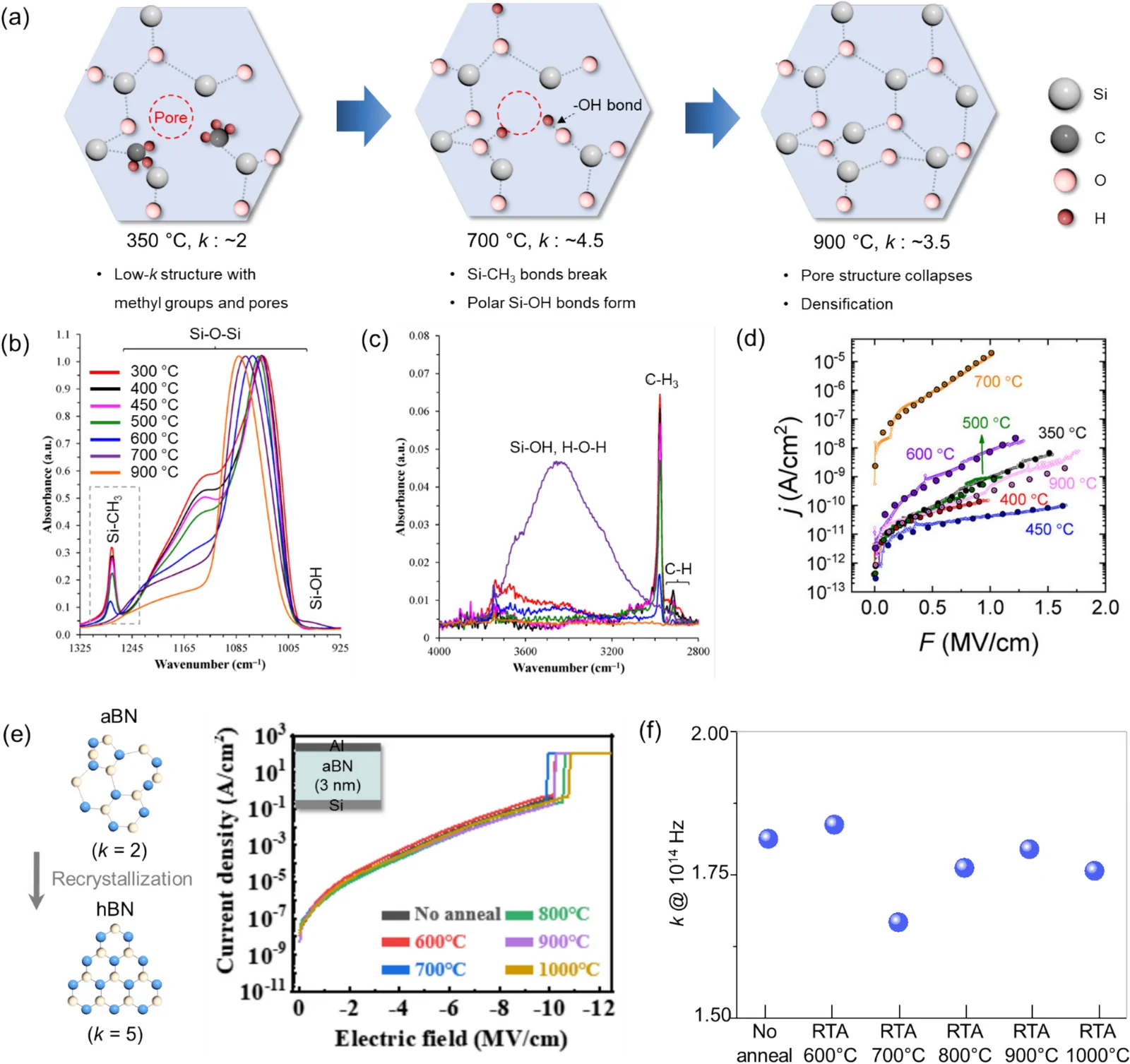
Thermally stable aBN as an alternative to thermally vulnerable porous low-*k* dielectrics. **a** Schematic illustration of thermal degradation in porous SiCOH during annealing. **b**, **c** FTIR analysis of annealed porous SiCOH, showing changes in **b** Si–CH_3_ bonding [57] and **c** Si–OH bonding [57]. **d** Annealing-temperature-dependent leakage current of porous SiCOH [57]. **e**, **f** Thermal stability of aBN after high-temperature annealing, showing stable **e** leakage current [25] and **f** low-*k* behavior [25]. **b**–**d** Adapted from ref. [57] under the terms of the Creative Commons Attribution License (CC BY 4.0) Copyright 2024, MDPI. **e** Adapted with permission from ref. [25]. Copyright 2023 IEEE

Thermal stability is another important requirement, because dielectric materials can be exposed to elevated temperatures during annealing and integration processes. However, porous SiCOH is thermally vulnerable. Annealing SiCOH at 700 °C leads to thermally induced chemical modification of the SiCOH network, where Si–CH_3_ groups are decomposed and more polar Si–OH bonding is generated (Fig. 5a–c) [57]. Because Si–OH bonds increase the polarizability of the dielectric matrix and promote moisture-related degradation, the *k* value increases and is accompanied by a substantial increase in leakage current of approximately four orders of magnitude (Fig. 5d) [57]. At 900 °C, Si–OH species can be removed; however, the porous network undergoes thermally driven densification because the pore surfaces tend to reduce their surface area and lower the total surface energy. As a result, pore collapse increases the film density and raises the *k* value relative to the initial state.

In contrast, aBN can maintain both low leakage current and a low-*k* value even after high-temperature annealing (Fig. 5e, f) [25]. This behavior is important because the low-*k* nature of aBN is associated with its amorphous bonding network. If aBN recrystallizes into an hBN-like ordered phase during thermal annealing, its dielectric constant could increase substantially, since crystalline hBN generally exhibits a much higher *k* value of approximately 5. This indicates that aBN does not readily recrystallize into an ordered phase during thermal annealing. The suppressed recrystallization can be related to the strong B–N bonding network and the high kinetic barrier for atomic rearrangement. Therefore, the key requirement for maintaining low-*k* is not merely the presence of an amorphous structure itself, but the thermal stability of the amorphous structure. In this respect, thermally stable aBN can be considered a promising alternative to porous low-*k* dielectrics.

## Future perspectives in low-*k* aBN dielectrics

### NAND cell reliability: hydrogen effect

**Fig. 6:**
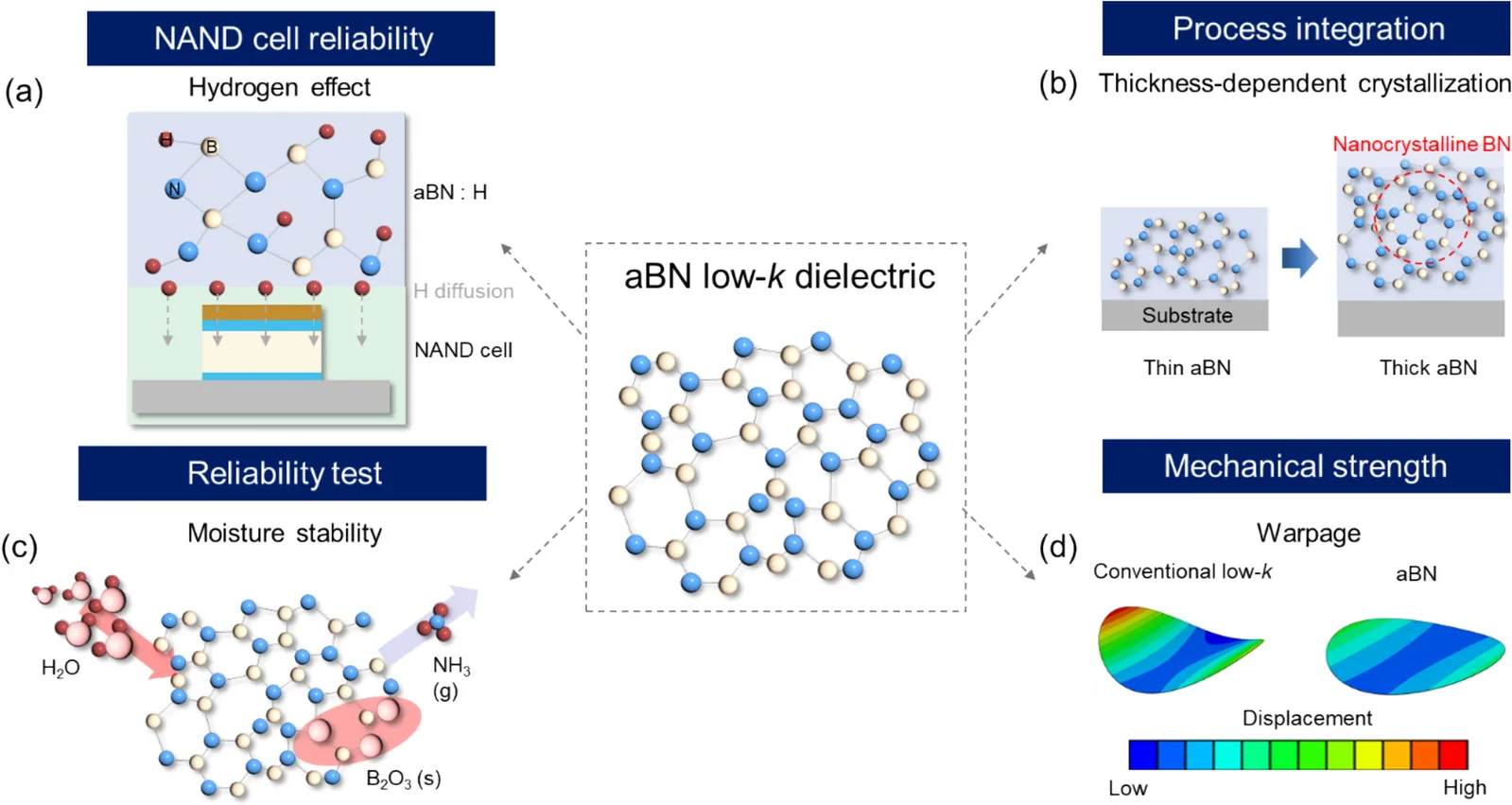
Toward a reliable aBN low-*k* dielectric for next-generation interconnects. **a** Schematic of hydrogen diffusion in a NAND cell. **b** Thickness-dependent crystallization of aBN, where thicker films can form nanocrystalline BN domains. **c** Possible moisture-induced degradation of aBN through water interaction with electron-deficient boron sites. **d** Wafer-warpage issue associated with residual film stress, highlighting the need for wafer-curvature evaluation of aBN

With the increasing demand for high-density memory, NAND flash technologies are moving toward vertically stacked architectures and multi-die package integration. In these highly stacked NAND structures, hydrogen introduced during fabrication, annealing, or post-treatment can diffuse through dielectric layers (Fig. 6a) [58]. Therefore, hydrogen incorporation should be considered a critical factor governing the electrical reliability of NAND flash memory devices. In SiN charge-trapping layers, hydrogen can exist as Si–H, N–H, or hydrogen-related defect complexes, which modify the density and energetic distribution of charge-trapping sites. Experimental studies have shown that hydrogen diffusion into nitride charge-trapping layers can induce trapped-electron loss through interactions with defect states, resulting in threshold-voltage shifts and retention degradation. These results indicate that hydrogen is not merely a residual impurity, but an active species that can modify charge storage and charge-loss mechanisms during device operation.

This hydrogen-related reliability issue is relevant to aBN because hydrogen incorporation can modify not only its defect states but also its local bonding structure and network connectivity. During aBN deposition, hydrogen can be incorporated in the form of B–H, N–H, or hydrogen-related defect complexes. Depending on its concentration and bonding configuration, hydrogen may alter the coordination environment, sp^2^/sp^3^ hybridization, network connectivity, and defect formation in aBN. As discussed in Sect. 3.2, moderate hydrogen incorporation can reduce low-coordinated sites and promote a denser, more highly connected sp^3^-rich network, thereby improving mechanical robustness. However, an increased sp^3^ fraction is also associated with a higher atomic density and higher *k*, indicating a trade-off between dielectric and mechanical properties. At moderate hydrogen contents, B–H and N–H bond formation may passivate low-coordinated B/N sites and suppress electrically active defects. This could reduce trap-assisted leakage pathways and improve dielectric reliability. However, excessive hydrogen incorporation may terminate the continuous B–N network, generate weakly bonded or low-coordinated sites, and introduce additional defect states. As a result, high hydrogen content could increase leakage current, lower breakdown strength, and accelerate time-dependent dielectric breakdown [59, 60]. Therefore, hydrogen concentration and bonding configuration should be optimized to simultaneously maintain a low-*k*, sufficient mechanical robustness, and electrical reliability.

If aBN is introduced as a capping layer, diffusion barrier, or bonding dielectric in NAND-related integration schemes, hydrogen-induced defect states influence local charge trapping and leakage current. Hydrogen diffusion near charge-trapping regions could contribute to threshold-voltage instability, memory-window variation, and retention loss. Therefore, hydrogen content should be considered a key parameter in aBN dielectric integration. Future studies should clarify how hydrogen concentration and bonding configuration affect the local sp^2^/sp^3^ bonding structure, *k*, mechanical robustness, leakage-current mechanisms, breakdown strength, TDDB behavior, and charge-retention stability of NAND cells. Such understanding will be essential for evaluating aBN as a reliable low-*k* dielectric material for NAND-related packaging and integration applications.

### Process integration: thickness-dependent crystallization

Although aBN shows high thermal stability, thickness-dependent crystallization remains an important issue (Fig. 6b) [25]. Ultrathin aBN can maintain low-*k* behavior, but as the film becomes thicker, nanocrystalline BN domains form and increase *k*. This behavior can be attributed to longer deposition times and reduced substrate constraints in thicker films, both of which promote structural ordering and bulk-like relaxation of the BN network. As a result, partial hBN-like ordering can occur, making it difficult to maintain the low-*k* characteristic. SiO_x_ insertion has been proposed as one strategy to suppress this crystallization [61]. By locally intermixing with the BN network, SiO_x_ can disrupt the continuity of the BN structure and inhibit hBN-like ordering. However, even when crystallization is suppressed, *k* remains higher than the IRDS target. Thus, retaining long-range disorder in the BN network is significantly important.

### Reliability tests: moisture stability

The moisture reliability of aBN remains unverified because Highly Accelerated Stress Test (HAST) data are still lacking. Because electron-deficient boron sites can interact with water molecules through Lewis acid–base interactions, aBN may be susceptible to hydrolysis or oxidation under humid conditions (Fig. 6c) [62]. Therefore, future studies should quantitatively compare the moisture stability of aBN with that of SiCOH or SiCN. For hybrid-bonding applications, additional reliability questions must also be addressed, including whether aBN can effectively suppress Cu oxidation, whether the Cu/aBN interfacial adhesion is sufficiently strong, and whether its moisture and thermal stability can be maintained during the hybrid bonding process.

### Warpage

Wafer warpage, defined as wafer bending induced by residual stress or coefficient of thermal expansion mismatch, is a key challenge in semiconductor manufacturing because excessive warpage can hinder lithography, bonding, and handling processes and cause mechanical failures such as cracking or interfacial delamination. To estimate the residual thin-film stress associated with wafer curvature, the Stoney equation is commonly used as a first-order approximation: [63, 64]

$$ \sigma_{{{\mathrm{internal}}}} = \left( {\frac{{E_{s} }}{{1 - v_{s} }}} \right)\frac{{t_{s}^{2} }}{{6t_{f} }}\left( {\frac{1}{{R_{f} }}\frac{1}{{R_{0} }}} \right) $$

where $E_{s}$, $v_{s}$,and $t_{s}$ are the Young’s modulus, Poisson’s ratio, and thickness of the substrate, respectively. $t_{f}$ is the film thickness, and $R_{f}$ and $R_{0}$ are the wafer curvatures after and before deposition of the thin film, respectively. This relation indicates that reducing the thickness of the deposited film can mitigate wafer warpage by lowering the bending moment imposed on the substrate.

For example, under a simplified blanket-film assumption, a 150 nm SiCN film with a residual stress of ~1300 MPa on a 780 µm Si substrate is estimated to induce wafer warpage on the order of 10^2^ µm for a 300 mm wafer [65]. In this regard, if aBN can be integrated as a dielectric or barrier layer several nanometers thick with comparable intrinsic stress, its contribution to wafer warpage would be much smaller than that of thicker SiCN-based capping or bonding layers (Fig. 6d). However, because BN-based films can also exhibit compressive stress depending on deposition conditions, quantitative wafer-curvature measurements are required to verify any warpage-related advantage of aBN.

### Wafer bonding

**Fig. 7:**
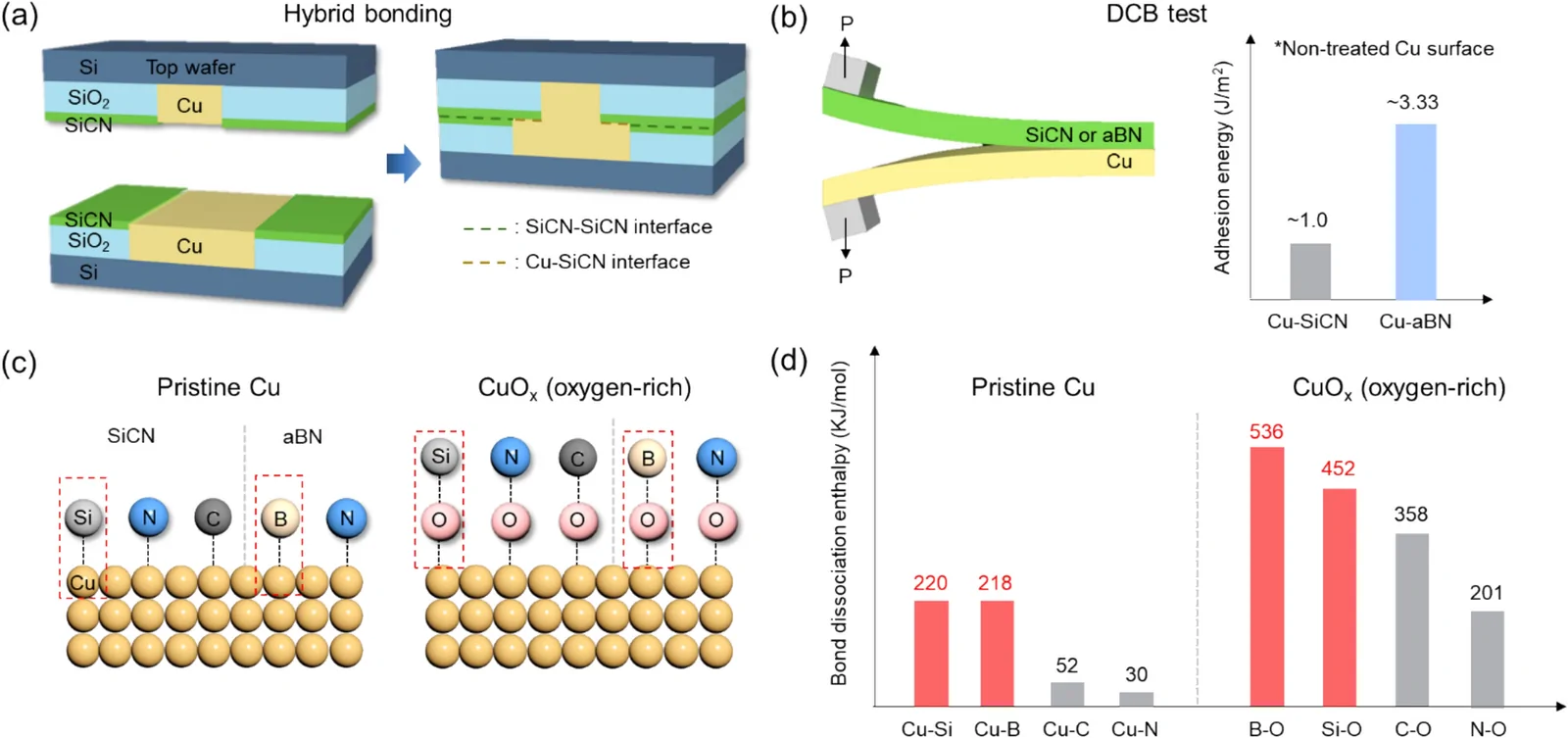
Potential application of aBN as an ultrathin bonding/interface dielectric for hybrid bonding. **a** Schematic illustration of conventional hybrid bonding based on SiCN–SiCN dielectric bonding layers. **b** Comparison of Cu–dielectric interfacial adhesion evaluated by double-cantilever beam testing. **c** Schematic comparison of interfacial bonding between aBN or SiCN and pristine Cu or partially oxidized Cu surfaces. **d** Comparison of bond dissociation enthalpies relevant to Cu/SiCN and Cu/aBN interfaces

Wafer bonding technologies, which integrate wafers or dies through interfacial bonding, have been developed to overcome the integration limits of memory devices. In hybrid-bonding schemes, SiCN–SiCN bonding layers have been widely investigated as dielectric bonding interfaces because they can provide Cu diffusion-barrier functionality and low-*k* characteristics (Fig. 7a) [66]. Continued technology scaling requires further reduction in chip thickness and interfacial dielectric thickness. However, the SiCN bonding layers used in hybrid-bonding processes are approximately 100–150 nm. If the SiCN layer is made too thin, it may no longer provide sufficient CMP process margin, dielectric isolation, film continuity, and Cu diffusion-barrier functionality. Therefore, scaling down the SiCN bonding-layer thickness creates a trade-off between thickness reduction and process/interfacial reliability.

If sufficient dielectric and barrier properties can be achieved at thicknesses of several nanometers, aBN could be considered as an ultrathin bonding/interface dielectric. Furthermore, aBN is expected to bond more strongly with Cu than SiCN, potentially suppressing interfacial delamination during hybrid-bonding integration. Cu–aBN interfaces have been reported to exhibit more than three times the adhesion energy of Cu–SiCN interfaces in double-cantilever beam (DCB) tests, suggesting that aBN may improve Cu–dielectric interfacial adhesion (Fig. 7b) [25, 67]. This enhanced adhesion may be understood in terms of interfacial bond dissociation enthalpy (Fig. 7c, d). On pristine Cu surfaces, Cu–B and Cu–Si bonds can have comparable bond strengths, so the adhesion difference between Cu–aBN and Cu–SiCN may be limited [68, 69]. However, Cu surfaces can be partially oxidized during a hybrid-bonding process. Under such partially oxidized Cu surface conditions, B–O-related bonding may form at the Cu/aBN interface, potentially leading to higher adhesion than Si–O-related bonding at Cu/SiCN interfaces. Therefore, aBN can be considered a promising alternative to SiCN bonding dielectrics. However, it should be emphasized that hybrid bonding with aBN remains unexplored. Therefore, aBN should be discussed as a promising candidate for ultrathin bonding or interfacial dielectric layers, supported by its low-*k* character, nonporous structure, strong Cu interfacial bonding, and Cu oxidation resistance.

## Conclusions

aBN represents a promising alternative IMD and bonding layer because of its dense nonporous structure, intrinsic low-*k* potential, reported metal diffusion-barrier capability, and possible interfacial adhesion advantages compared with conventional dielectric layers. However, further studies on process-dependent dielectric properties, interfacial bonding strength, moisture stability, residual stress, Cu oxidation suppression, and patterned-structure reliability are required before product-level implementation can be considered. Nevertheless, these unresolved issues also highlight the strong research value of aBN as a next-generation dielectric/interface material for advanced BEOL interconnects.

## Data Availability

No datasets were generated or analyzed during the current study.
